# Oral Burkitt's Lymphoma - Case Report

**DOI:** 10.1016/S1808-8694(15)30583-8

**Published:** 2015-10-19

**Authors:** Roseana de Almeida Freitas, Simone Souza Lobão Veras Barros, Lêda Bezerra Quinderé

**Affiliations:** 1 Doctor in oral pathology. Professor of the graduate program in oral pathology, UFRN.; 2 Doctoral student in the oral pathology course, UFRN. Professor of oral pathology, part of the dentistry course, UFPI.; 3 Doctor in oral pathology. Professor of pediatric dentistry in the dentistry course, UFRN. Graduate program in oral pathology (Universidade Federal do Rio Grande do Norte - UFRN)

**Keywords:** oral cancer, burkitt's lymphoma, oral lymphoma

## Abstract

Burkitt's lymphoma is a poorly differentiated rare and aggressive type of non-Hodgkin's lymphoma. This article reports the case of a male child aged seven years, who was examined at the Odontopediatric Clinic of the UFRN Dentistry Department. The patient presented a tumor in the premolar region of the mandible; teeth were mobile in this region. Radiology revealed a diffuse radioluscent area which was diagnosed histopathologically as Burkitt's lymphoma. The patient was treated with polychemotherapy; complete remission of the disease was attained.

## INTRODUCTION

Burkitt's lymphoma is a highly aggressive non-Hodgkin lymphoma that has the highest cell proliferation rate among human neoplasms.^1^, ^2^ It occurs predominantly in the first decades of life, mostly in males, and with significant affinity for gnathic bones, especially the maxilla.^3^, ^4^ This tumor may progress very rapidly in the mouth, presenting as a facial tumor or an exophytic mass involving the maxillary bones.^5^ The purpose of this paper was to report a case of Burkitt's lymphoma in a 7-year-old child, emphasizing the clinical features, radiographic findings and the histopathology of this rare lesion.

## REVIEW OF THE LITERATURE AND THE DIFFERENTIAL DIAGNOSIS

Burkitt's lymphoma is a rare poorly differentiated lymphocytic lymphoma characterized by monoclonal proliferation of B-lymphocytes.^5^ Cytogenetically, there is rearrangement of the C-myc oncogene, which is characterized by the presence of typical translocations: t (8; 14) (q24; q32) or their rare variants: t (8; 22) (q24; q11) or t(2; 8) (q12; q24).^6^, ^7^

Various studies have strongly suggested an association between the Epstein-Barr virus (EBV) and the pathogenesis of Burkitt's lymphoma. DNA sequences of this virus may be found in B cells, and elevated anti-EBV antibodies are found in patients with Burkitt's lymphoma.^3^, ^4^, ^6^ The EBV inhibits programmed cell death and helps develop and maintain Burkitt's lymphoma.^8^

Clinically, this disease occurs mostly in children. The incidence peaks between ages 3 and 8 years, and males are affected about twice as frequently as females. Lesions involve mostly the maxilla, the mandible and the abdomen. The most frequent signs of this disease in the mouth are local tumors and altered tooth mobility. Symptoms are sparse, consisting of local pain, tenderness and parestesia.^2^, ^4^, ^5^

Ardekian et al.^5^ reviewed the clinical features of 13 Burkitt's lymphoma cases, and found that eight patients were male, that the mean age was 15.3 years, and that the maxilla was the most commonly affected site. Boerma et al.^1^ found a bimodal age distribution, with a first incidence peak between 6 and 10 years and a second incidence peak after age 60 years. Ukboko et al.^9^ found that 51.2% of Burkitt's lymphomas occurred in maxillary bones. Nakagawa et al.^10^ found 18 cases of Burkitt's lymphoma in a sample of 95 non-Hodgkin's lymphoma cases; the tumor site was the head and neck in five of the 18 cases.

Radiographic findings in Burkitt's lymphoma include radiolucent images of bone destruction with poorly defined and irregular margins.^2^, ^4^ There is a “starry sky” microscopic aspect with small, diffusely proliferated, monomorphic, immature and undifferentiated lymphocytes interspaced by numerous macrophages within abundant cytoplasm.^5^, ^7^

Burkitt's lymphoma is treated preferentially with intensive chemotherapy; 5-year survival rates range between 75 and 95%, depending on the stage of the lesion at the time of diagnosis.^3^, ^4^

The differential diagnosis should be made with the following conditions: per apical lesions, ameloblastoma, other non-Hodgkin's lymphomas, undifferentiated carcinomas and sarcomas, and leukemia.^2^, ^5^

## CASE REPORT

J.M.M.S., a white male patient aged 7 years, presented at the Pediatric Dentistry Clinic of the UFRN Dental School. His mother reported that the right region of the mandible body was increased in size. She also stated that the patient had been seen by a dental surgeon, who had suggested the possibility of a dentoalveolar abscess, and had started antibiotic therapy; the clinical picture remained unchanged 17 days later, after which the dental surgeon removed dental elements 84 and 85, which were mobile. The extra-oral physical examination showed that the right mandible was in fact increased in size. The intra-oral physical examination revealed an asymptomatic tumor-like mass located in the vestibular portion of the right aspect of the mandible body. On radiology, a diffuse radiolucent area was seen in the region of the abovementioned teeth. An incision biopsy was done, leading to a histopathological diagnosis of Burkitt's lymphoma. The patient was referred to the Oncology Unit of the Varela Santiago Children's Hospital (Hospital Infantil Varela Santiago); polychemotherapy was undertaken and was successful. Seven years later, the patient is disease free and shows no signs of recurrence of metastasis.

**Figure 1:**
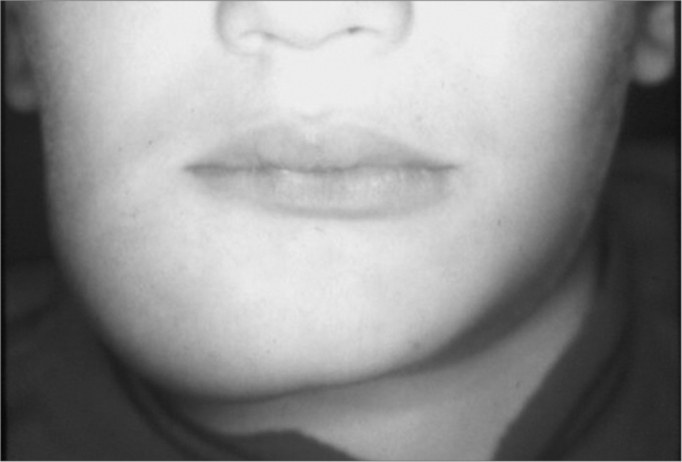
Burkitt's lymphoma - Extra-oral clinical aspect - facial tumor on the right.

**Figure 2:**
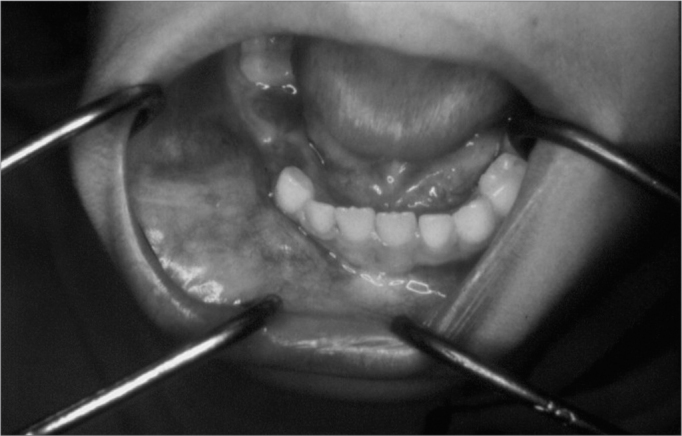
Burkitt's lymphoma - Intra-oral clinical aspect - an asymptomatic tumor-like mass on the mandible premolar area.

**Figure 3:**
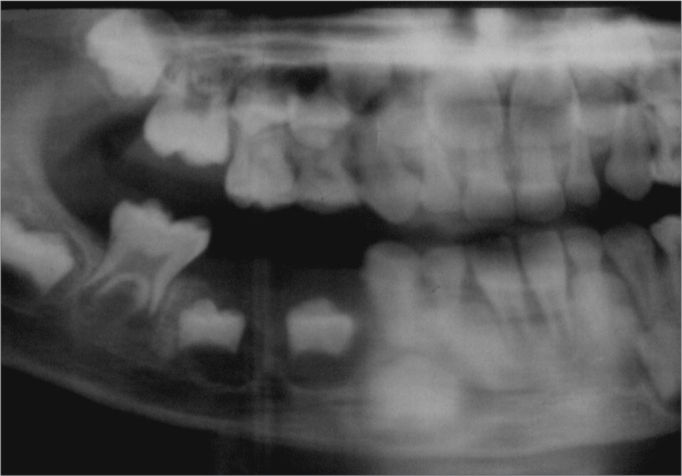
Burkitt's lymphoma - Panoramic radiograph - a diffuse translucid area is seen on the mandible premolar area.

## DISCUSSION

Burkitt's lymphoma is a rare and rapidly progressive tumor that occurs in an early differentiation stage of B cells.^3^

In this case, Burkitt's lymphoma occurred in the mandible of a child aged 7 years. The mandible is one of the most common sites for this disease;^2^, ^4^, ^5^ the patient in question was within the peak incidence age for Burkitt's lymphoma.^2^, ^4^, ^5^ Clinical findings (increased volume of the face, presence of an intra-oral mass and tooth mobility) and the radiology (poorly defined borders of the lesion) of this case are among the most commonly reported features of Burkitt's lymphoma in the literature;^2^, ^4^, ^5^ these findings, however, may be encountered in many other conditions. The definitive diagnosis of Burkitt's lymphoma was made in the histopathological exam of an incision biopsy fragment, after which the patient was referred for oncological therapy.

**Figure 4:**
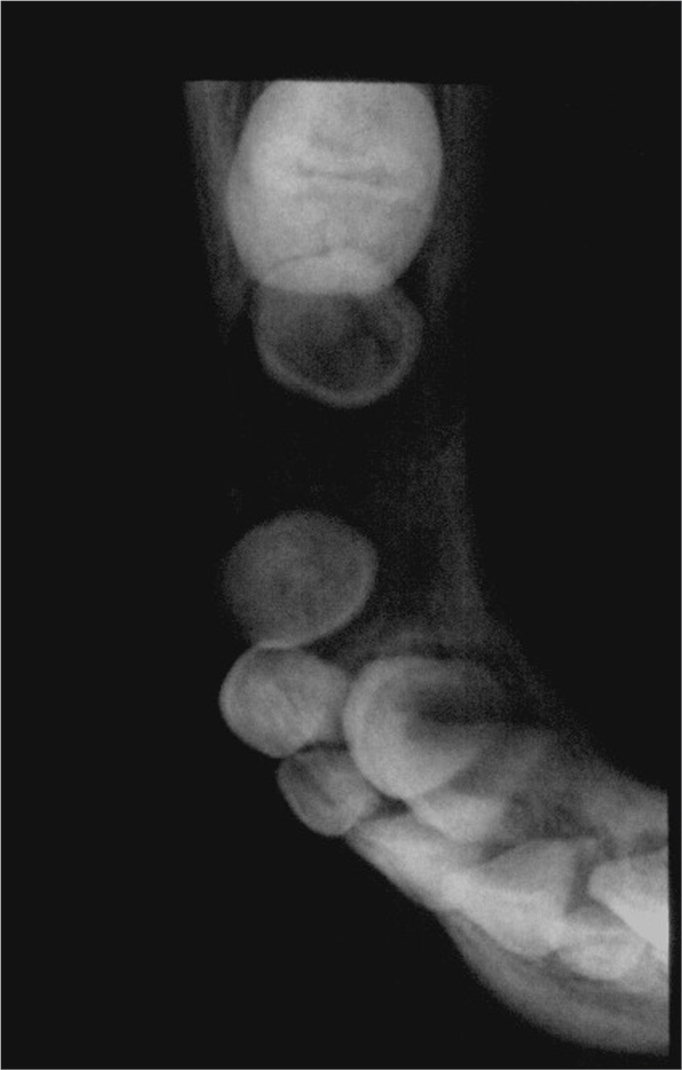
Burkitt's lymphoma - Occlusal radiograph - note dislocation of the premolar germs and rupture of the cortical layer.

**Figure 5:**
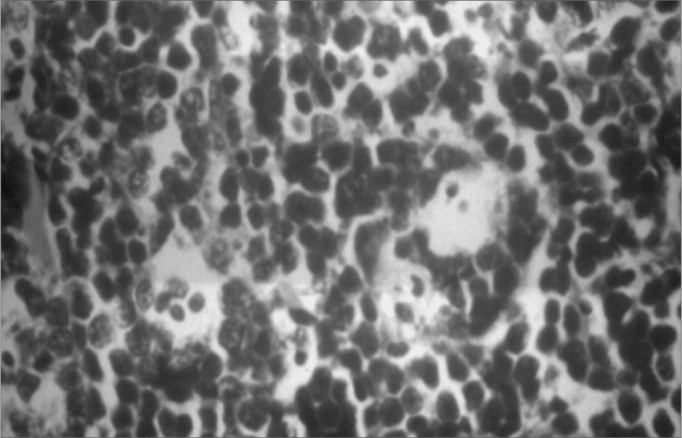
Burkitt's lymphoma - Histology - note pleomorphic lymphocytes and macrophages with a clear cytoplasm containing cell remains.

**Figure 6:**
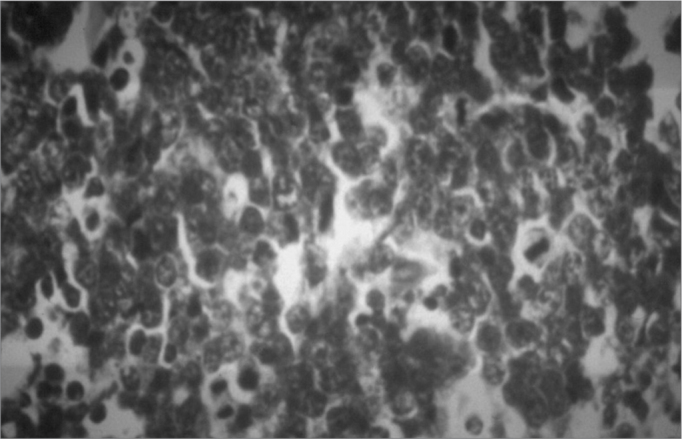
Burkitt's lymphoma - Histology - detail showing mitosis.

**Figure 7:**
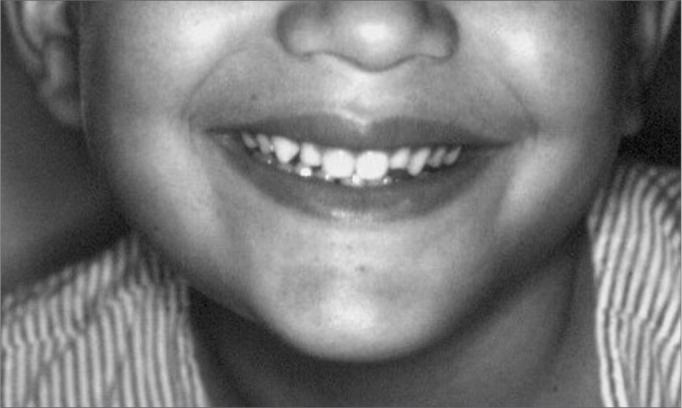
Burkitt's lymphoma - During polychemotherapy - extra-oral findings after the second cycle of polychemotherapy.

**Figure 8:**
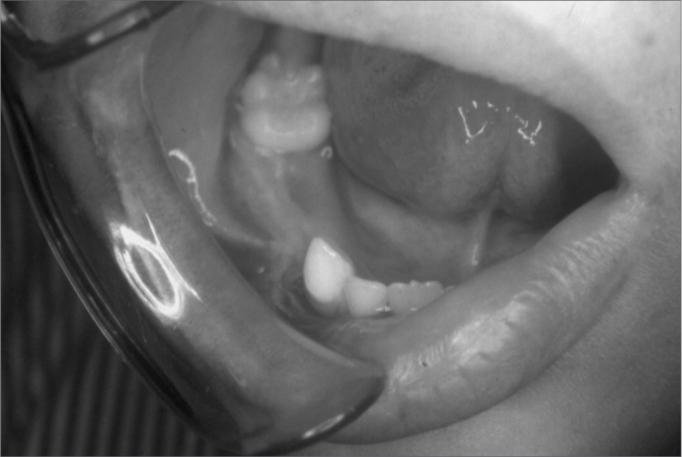
Burkitt's lymphoma - During polychemotherapy - Intra-oral findings after the second cycle of polychemotherapy.

Burkitt's lymphoma responds well to chemotherapy.^2^, ^3^, ^4^ In this case the lesion regressed completely; there are no signs of recurrence or metastasis 7 years following chemotherapy.

## FINAL COMMENTS

Burkitt's lymphoma is a rare neoplasm that has affinity for the maxillary bones, especially in patients within the first decade of life. This disease should be considered in the diagnosis of rapidly growing lesions located in the maxilla or the mandible, which progress to sizeable tumor-like masses, associated with tooth mobility and poorly defined borders on radiographic exams. Histopathology provides the conclusive diagnosis.
